# Advances in the study of Ophiopogon japonicus polysaccharides: structural characterization, bioactivity and gut microbiota modulation regulation

**DOI:** 10.3389/fphar.2025.1583711

**Published:** 2025-05-02

**Authors:** Jiani Li, LiQuan Zhou, ZuoWei Xiao

**Affiliations:** ^1^ Homologous Innovation Laboratory of medicine and food, Hunan University of Chinese Medicine, Changsha, China; ^2^ Hunan Engineering and Technology Research Center for Health Products and Life Science, Hunan University of Chinese Medicine, Changsha, China; ^3^ School of Pharmacy, Hunan University of Chinese Medicine, Changsha, China

**Keywords:** Ophiopogon japonicus polysaccharides, Structure, biological activities, antidiabetic activity, gut microbiota

## Abstract

Ophiopogon japonicus polysaccharides (OJPS), the principal bioactive constituents isolated from Ophiopogon japonicus, demonstrate substantial physiological efficacy. OJPS is characterized by a high molecular weight, typically ranging from 2.48 to 324.7 kDa. Emerging evidence indicates that OJPS modulates the composition and structural organization of the gut microbiota, thereby maintaining intestinal barrier integrity and enhancing both gastrointestinal and systemic homeostasis. Moreover, OJPS and its metabolic derivatives engage in dynamic interactions with microbial communities, mediating cellular signaling cascades and endocrine regulation to elicit hypoglycemic effects. Despite these findings, comprehensive analyses of OJPS extraction and purification methodologies, structural elucidation, biological functionalities, and mechanistic insights into its crosstalk with the gut microbiota remain scarce. This review systematically synthesizes contemporary knowledge pertaining to the preparation, structural attributes, bioactivity, and mechanistic underpinnings of OJPS, with particular emphasis on its dual regulatory role in host physiology and gut microbial ecology.

## 1 Introduction

Ophiopogon japonicus (L. f.) Ker-Gawl., a perennial herbaceous species of the family Liliaceae, is pharmacologically characterized by its desiccated fleshy tuberous roots, which have been extensively employed in traditional medicine. Predominantly distributed across China, this species is widely regarded as one of the most esteemed medicinal plants in Chinese pharmacopeia. Initially documented in the classical treatise Shennong Ben Cao Jing, it has been historically utilized to alleviate cardiac fire, replenish yin, hydrate pulmonary tissues, and stimulate fluid secretion. In 2024, Ophiopogon japonicus was formally incorporated into China’s Catalog of Medicinal and Food Homologous Substances, thereby expediting its application in patent Chinese medicines and functional food products. Phytochemical investigations have elucidated a diverse array of bioactive metabolites in Ophiopogon japonicus, including steroidal saponins, isoflavones, terpenoids, and polysaccharides. Among these, Ophiopogon japonicus polysaccharides (OJPS) constitute a principal bioactive fraction, which has been demonstrated to exhibit immunomodulatory, antioxidant, cardioprotective, cerebrovascular-protective, and hypoglycemic activities (Wang Yang, 2023).

As the principal bioactive macromolecule, OJPS are structurally characterized by a backbone predominantly composed of β-fructose and α-glucose residues linked via glycosidic bonds. The gut microbiota serves as a critical mediator between polysaccharides and host physiological homeostasis. Plant-derived polysaccharides not only function as essential carbon and energy substrates for intestinal microbial communities but also contribute to the maintenance of intestinal barrier integrity. Microbial fermentation of polysaccharides yields short-chain fatty acids (SCFAs), which exert regulatory effects on the intestinal microenvironment. Accumulating evidence from recent studies has substantiated the regulatory role of OJPS in gut microbiota modulation. OJPS is predominantly metabolized by gut microbiota, and its bioactive metabolites have been demonstrated to modulate microbial composition and diversity while concurrently mediating systemic beneficial effects through microbiota-derived signaling pathways. Thus, elucidating the mechanistic interplay between OJPS and intestinal microbiota is essential for understanding their synergistic roles in preserving intestinal homeostasis and promoting systemic health.

In recent years, several comprehensive reviews have been published elucidating the research progress regarding the extraction, isolation, structural characteristics, and biological activities of OJPS. Zhu et al. (2025) systematically documented the preparation methodologies, structural configurations, pharmacological properties, and potential therapeutic applications of OJPS. Concurrently, Zhang et al. (2024) conducted a rigorous evaluation of the extraction protocols, purification techniques, structural elucidation, and chemical modifications of OJPS. Notably, while these reviews have extensively covered the methodological aspects of OJPS processing and its fundamental bioactivities, the intricate relationship between OJPS and gut microbiota remains insufficiently explored. Current literature demonstrates a significant research gap in understanding the microbiota-mediated mechanisms through which OJPS exerts its physiological effects.

Consequently, this review comprehensively evaluates current research progress regarding the extraction, purification, structural characterization, and biological activities of Ophiopogon japonicus polysaccharides (OJPS), with particular emphasis on elucidating their microbiota-mediated effects on human health. The intricate interactions between OJPS and gut microbiota are critically examined, and the underlying mechanisms through which gut microbiota maintain host homeostasis are systematically analyzed. This review is expected to expand the utilization and development of Ophiopogon japonicus resources, provide a reference for the treatment of diseases related to the gut microbiota, and provide information for drug and food development.

## 2 Extraction and purification

The extraction of OJPS is predominantly performed through aqueous ethanol precipitation, a method that effectively eliminates impurities via alcohol sedimentation while maintaining satisfactory extraction accuracy. However, this conventional approach is constrained by several limitations, including prolonged processing duration, suboptimal extraction efficiency, and excessive energy requirements. With the development of energy saving, environmental protection, high efficiency, and sustainability, various new and efficient extraction methods have emerged, including enzyme-assisted extraction, microwave-assisted extraction, and ultrasound-assisted extraction. However, these methods still have problems that need to be addressed; for example, in microwave-assisted extraction, there are problems such as solvent evaporation and uneven temperature rise, which affect the efficiency and stability of extraction. Enzyme-assisted extraction, owing to enzyme specificity and instability, makes the extraction process of OJPS more complex and unstable. Similarly, although ultrasound-assisted extraction can significantly increase the extraction rate of polysaccharides, it can possibly alter the glycosidic bond and affect biological activity. With the advancement of science and technology and increasing research on OJPS, more new methods will be more widely applied to the extraction of OJPS, which will further improve efficiency and sustainability. The combined use of various extraction methods is a new approach for polysaccharide extraction (Table 1).

**TABLE 1:** Different extraction methods of OJPS.

Methods	Principle	Extraction conditions	Extraction rate	Evaluation	References
Hydrotropic alcohol precipitation	Utilizes water as a solvent to disrupt cell walls, thereby dissolving and isolating polysaccharides	Liquid-solid ratio = 8 ∶ 1 (mL/g),t = 1.7 h,T = 80°C,Amount of 95% ethanol solution 65 mL	20.17%	Low cost, simple operation, safe reagents, but long time required, high energy consumption	Wang Ying (2019)
		Liquid-solid ratio = 20∶1 (mL/g),t = 135 min,T = 90°C,Amount of 85% ethanol solution 60 mL	26.93%		Zhang Xue-Lian (2022)
		Liquid-solid ratio = 9 ∶ 1 (mL/g) repeat three times, 80% ethanol solution	10.02%		Zhang Jing (2021)
		Liquid-solid ratio = 10 ∶ 1 (mL/g),t = 4 h,T = 100°C, 95% ethanol solution	10%		Wang et al. (2019a)
Enzyme extraction	Enzyme treatment can destroy the cell wall or cleave the macromolecular structure, reduce the extraction resistance and increase the polysaccharide yield	Liquid-solid ratio = 35:1 (mL/g),pH = 5.0, T = 45°C,t = 120 min,Enzyme dosage 0.5%	4.12%	High efficiency, mild reaction conditions,low energy consumption, but high cost, poor enzyme stability, easy inactivation	Huang Shan (2009)
Microwave extraction	The penetrating and selective heating of microwave can quickly destroy the cell structure and promote the dissolution of polysaccharides	Liquid-solid ratio = 40:1 (mL/g), t = 20 min,T = 40°C	13.8%	Even heat distribution, short time, but high energy consumption, high cost	Lu Shaoling (2018)
		Liquid-solid ratio = 60:1 (mL/g), t = 20 min, P = 700 w	8.20%		Xiang (2007)
Ultrasound extraction	The high-frequency vibration and cavitation of ultrasound can destroy cell structure and enhance solvent permeability and mass transfer efficiency	Liquid-solid ratio = 20: 1 (mL/g),T = 41°C, t = 51 min,P = 250 W	20. 98%	High efficiency, short time, low energy consumption, but the required cost is high, high operation requirements	Feng Si-Si (2024)
		Liquid-solid ratio = 22:1 (mL/g), t = 27 min, P = 200 W	39.49%		Wen and Su (2023)
		Ultrasonic conditions were: 240 W ultrasonic treatment lasting 10 s, with an interval of 15 s; repeated 90 times	8.74%		Wang et al. (2018)
		Ultrasonic condition: 80 W ultrasonic treatment for 10 s, with an interval of 15 s; repeated 90 times	11.87%		Wang L. Y. et al. (2012)
Ultrasound-assisted dual-phase aqueous extraction	Extraction and separation are based on the difference in solubility of substances in two immiscible phases.Ultrasound-assisted extraction, on the other hand, damages the cellular structure of the plant and enhances solute dissolution and diffusion through the technique of “cavitation”	Liquid-solid ratio = 17: 1 (mL/g),ethanol concentration of 34%, ammonium sulfate concentration of 23%, P = 480 W	9.41% ± 0.12%	Low cost, high efficiency, can significantly improve the purity of polysaccharides	Tian et al. (2024)

The extraction of OJPS through conventional methods frequently yields products contaminated with significant quantities of proteinaceous material, chromophoric compounds, and flavonoid derivatives. These impurities not only adversely affect the purity of the isolated polysaccharides but also substantially interfere with accurate structural characterization and reliable bioactivity evaluation. Protein removal represents a particularly critical step in the purification process of polysaccharides.

To obtain OJPS of pharmaceutical-grade purity suitable for detailed analytical investigations, an optimized purification protocol was systematically developed, incorporating sequential decolorization, deproteinization, and dialysis procedures. The decolorization process merits particular attention, as residual pigment molecules have been shown to artificially elevate molecular weight determinations through spectroscopic interference while simultaneously creating steric hindrance that obstructs cellular uptake pathways and consequently diminishes biological activity. Currently employed deproteinization techniques include the Sevage method, enzymatic digestion approaches, trichloroacetic acid precipitation, and combined enzyme-Sevage treatment. Recent comparative investigations have conclusively demonstrated that the integrated enzyme-Sevage method achieves significantly enhanced protein removal efficiency compared to individual techniques (Zhang et al., 2020), suggesting potential synergistic interactions between enzymatic and chemical purification mechanisms that warrant further investigation.

Further purification of OJPS forms the basis for exploring its chemical structure and biological activity. This purification stage employs physicochemical techniques guided by the molecular weight distribution, surface charge properties, and structural features of the polysaccharide to enhance purity. The implementation of established methodologies such as fractional precipitation, ultrafiltration membrane separation, ion-exchange chromatography, and size-exclusion chromatography enables selective isolation of target polysaccharide fractions. Owing to the complexity of the polysaccharide molecular structure, it is difficult to choose an appropriate purification method to obtain highly homogeneous polysaccharides. Stepwise precipitation is a method that uses ethanol to precipitate polysaccharides in several gradients according to the principle of “similar solubility” to achieve purification. This method is typically used for large-scale purification of polysaccharides. However, this method may lead to intra- and intermolecular self-assembly, resulting in changes in molecular weight and morphological features, as well as changes in chain conformation and biological activity, making the structural analysis of polysaccharides more difficult (Wang H. Y. et al., 2019). Membrane separation technology is characterized by high separation efficiency and high applicability. For example, OJPS maximally retains the original active metabolites after ceramic membrane ultrafiltration and its purification effect is better than that of high-speed centrifugation and direct decompression concentration (Li Jing, 2017). In addition, column chromatography is a commonly used method in the separation of OJPS, such as gel chromatography, anion-exchange chromatography, and macroporous resin column chromatography. Column chromatography has high separation efficiency and stable performance and is suitable for the separation of OJPS with different molecular weights. In gel chromatography column chromatography, commonly used gels include Sephadex and Sepharose, which utilize the principle of size exclusion and varying concentrations of salts and buffer solutions as eluents to purify some of the proteins and pigments in adsorbed OJPS In addition, column chromatography is a commonly used method in the separation of OJPS, such as gel chromatography, anion-exchange chromatography, and macroporous resin column chromatography. Column chromatography has high separation efficiency and stable performance and is suitable for the separation of OJPS with different molecular weights. Subsequently, in gel chromatography column chromatography, commonly used gels include Sephadex and Sepharose, which utilize the principle of size exclusion and varying concentrations of salts and buffer solutions as eluents to purify some of the proteins and pigments in adsorbed OJPS (Wen and Su, 2023).

Consequently, the development of an optimized purification system that ensures both high efficiency and reproducibility while minimizing procedural variability represents a critical research priority. Such advancement is essential to eliminate potential confounding factors that may interfere with the accurate structural elucidation of OJPS and subsequent biological activity assessments.

## 3 Structural characterization

Polysaccharides exhibit complex and distinctive structural configurations that fundamentally govern their biological activities and functional properties. The biological potency of polysaccharides is determined by multiple structural parameters, including monosaccharide composition, glycosidic linkage patterns, and molecular weight (Mw) distribution. OJPS demonstrate a broad Mw range of 2.48-324.7 kDa (Table 2), typically presenting as high-molecular-weight polymers with considerable polydispersity. Structural analyses reveal that OJPS primarily consist of β-(1→4)-linked glucose and β-(1→3)-linked fructose residues in the main chain, featuring acetyl modifications and rhamnose branch points. This structural heterogeneity not only influences fundamental physicochemical characteristics (e.g., aqueous solubility and solution viscosity) but also directly modulates biological activity. Monosaccharide composition analyses indicate the predominant presence of glucose (Glc) and fructose (Fru), with minor constituents including arabinose (Ara), galactose (Gal), and mannose (Man). Certain OJPS fractions may additionally contain rhamnose (Rha) residues. Notably, acetylated OJPS fractions have been demonstrated to exhibit enhanced macrophage-stimulating capacity, while non-acetylated counterparts are more susceptible to microbial degradation into bioactive oligosaccharides within the gastrointestinal tract (Gu et al., 2018). Comparative studies have revealed significant structural variations between OJPS preparations obtained through different extraction methodologies. For instance, ultrasonically extracted POJ-U1a displays distinct structural characteristics compared to its hot water-extracted counterpart, likely attributable to ultrasound-induced glycosidic bond cleavage (Wang L. Y. et al., 2012). Geographical variations significantly impact OJPS composition, as evidenced by phytochemical analyses. Chen et al. (2010) reported modest variations in polysaccharide content and monosaccharide profiles across different cultivation regions, harvest years, and Ophiopogon subspecies. Subsequent investigations by Xin Ya et al. (2024) systematically compared nine geographical sources, revealing consistently higher polysaccharide yields in Zhejiang-origin specimens relative to Sichuan-derived materials. While the fructose-to-glucose molar ratios remain comparable (approximately 15:1 vs. 14:1, respectively) between these regional variants (Chen et al., 2022), Zhejiang OJPS demonstrates superior antioxidant and immunostimulatory activities. This enhanced bioactivity may be attributed to differential contents of minor monosaccharides (Man, Ara, and Xyl), which have been correlated with antioxidant capacity and immunomodulatory potential. Further investigation is required to elucidate the structure-activity relationships governing these pharmacological effects and to establish quantitative correlations between physicochemical properties and biological responses.

**TABLE 2:** Chemical composition of OJPS.

Name	Molecular weight (kDa)	Monosaccharide composition	Molar ratio (nmol)	Method	Refences
MDG-1	4.8	Fru,Glc	35:1	HPLC,UV,1H NMR,13C NMR	Xu et al. (2005)
AP-1	124.3	—	—	HPGPC	Huang Ni (2011)
AP-2	324.7	—	—	HPGPC	Huang Ni (2011)
AP-3	6.7	—	—	HPGPC	Huang Ni (2011)
OJPS-2-SG	125	Man,Gal,Glc,Rha	5.15:26.39:8.7284	HPGPC,UV,FT-IR	Tian et al. (2024)
OJP2	35.2	Rha,Ara,Xyl,Glc,Gal	0.5:5:4:1:10	HPGPC,GC-MS	Fan Y. P. et al. (2015)
OPF-1	48	Fru,Glc	16:1	HPLC,GC,IR	Lv et al. (2012)
OJP1	35.2	Ara,Glc,Gal	1:16:8	HPGPC,GC	Chen et al. (2010)
POJ-U1b	4.02	Glc		GC,FT-IR,1H NMR,13C NMR	Wang X. M. et al. (2012)
OJP-W1	2.48	Fru,Glc	17:1	HPGPC,1H NMR,13C NMR,FT-IR	Wang et al. (2019b)
OJP 1	35.2	Ara,Glc,Gal	1:16:8	GC,HPGPC	Chen et al. (2013)
ROH05	16.7	Gal		HPGPC,RID,UV,GC-MS,1H NMR 和 13C NMR	Gu et al. (2018)
LSP	4.742	Glc,Fru	28:1	GC,GC-MS,infrared, NMR	Gong et al. (2017)
OJP	4.925	Glc,Fru	29:1	GC,GC-MS,infrared, NMR	Gong et al. (2017)
LMP	4.138	Glc,Fru	24:1	GC,GC-MS,infrared, NMR	Gong et al. (2017)
ORP-1	3.667	Glc,Fru	0.85 ∶ 0.15	HPGPC,IC,FT-IR,NMR	Wang et al. (2023)
OJZ	—	Fru,Glc,Ara,Man,Gal,Xyl	93.65:6.48:1.00:0.88:0.76:0.39	HPSEC-MALLS-RID,HPAEC-PAD	Chen et al. (2022)
OJC	—	Fru,Glc,Ara,Man,Gal,Xyl	175.10:12.83:1.00:1.21:1.08:0.37	HPSEC-MALLS-RID,HPAEC-PAD	Chen et al. (2022)
LM	—	Fru,Glc,Ara,Man,Gal,Xyl	253.30:21.98:1.00:0.65:0.58:0.33	HPSEC-MALLS-RID,HPAEC-PAD	Chen et al. (2022)
LS		Fru,Glc,Ara,Man,Gal,Xyl	236.06:17.31:1.00:0.89:0.64:0.35	HPSEC-MALLS-RID,HPAEC-PAD	Chen et al. (2022)
POJ	19.315 KDa	Glc, Man, Gal,Xyl	4.35:5.21:1.34: 1	PMP-Precolumn Derivatization、infrared	Ren et al. (2022)

## 4 Biological activities of OJPS

### 4.1 Hypoglycemic activity

Diabetes mellitus (DM) represents a heterogeneous group of metabolic disorders characterized by persistent hyperglycemia, with growing evidence indicating that OJPS possess considerable therapeutic potential against diabetes and its associated complications (He et al., 2023). Type 2 diabetes mellitus (T2DM), primarily driven by insulin resistance, manifests as systemic dysregulation of carbohydrate, lipid, and protein metabolism. Experimental investigations have demonstrated that OJPS administration effectively mitigates organ damage in diabetic models through dual mechanisms of reducing oxidative stress markers including malondialdehyde (MDA) and lipid peroxidation products while simultaneously enhancing endogenous antioxidant defense systems (Ding et al., 2025). The antidiabetic effects of OJPS are further mediated through improvement of insulin sensitivity via modulation of the InsR/IRS-1/PI3K/Akt/GSK-3β/Glut-4 signaling pathway, resulting in significant amelioration of characteristic metabolic abnormalities including hyperglycemia, hyperinsulinemia, and dyslipidemia in diabetic KKAy mice (Wang X. M. et al., 2012). It should be emphasized that while the KKAy murine model serves as a valuable tool for investigating obesity-associated diabetes, its translational relevance to human T2DM may be limited due to inherent differences in disease progression and pathophysiology. The metabolic phenotype of this spontaneous polygenic model is intrinsically shaped by progressive obesity development, potentially restricting its utility for studying advanced diabetic complications. Notably, OJPS has been shown to confer pancreatic β-cell protection through multiple mechanisms. Comprehensive studies by Mao et al. (2020) revealed that OJPS enhances glucose-stimulated insulin secretion in pancreatic β-cells while concurrently suppressing IL-1β-mediated inflammatory responses through inhibition of the IKK-NF-κB pathway. These beneficial effects were consistently observed across both genetic (db/db) and dietary (high-fat diet-induced) murine models of diabetes, accompanied by significant preservation of functional β-cell mass. The observed variability in therapeutic efficacy among different OJPS preparations likely stems from extraction method-dependent structural heterogeneity, suggesting the existence of important structure-activity relationships that merit further investigation to optimize therapeutic potential.

Furthermore, OJPS has been demonstrated to exert therapeutic effects on diabetic nephropathy and gestational diabetes mellitus through multiple mechanisms, primarily via attenuation of chronic hyperglycemia and reduction of serum albumin concentrations (Wang et al., 2015). Experimental evidence indicates that specific polysaccharide fractions derived from Ophiopogon japonicus effectively suppress cardiomyocyte apoptosis in diabetic atherosclerotic rabbit models. This cardioprotective effect is mediated through modulation of critical signaling pathways, including downregulation of the AGE-RAGE axis and regulation of apoptosis-related proteins such as JNK, caspase-3, and Bcl-2 (Jin et al., 2020).

### 4.2 Antioxidant activities

The current landscape of antioxidant therapeutics is predominantly dominated by synthetic compounds, which are frequently associated with undesirable side effects. This limitation has prompted growing scientific interest in the exploration of naturally derived antioxidants, which are increasingly recognized for their diverse biological sources, favorable safety profiles, and potent biological activity (Lei et al., 2021). Ophiopogon japonicus polysaccharides (OJPS) have been demonstrated to exhibit remarkable antioxidant properties through multiple mechanisms. Experimental studies have revealed that OJPS effectively scavenges reactive oxygen species, including hydroxyl radicals, superoxide anions, and 1,1-diphenyl-2-picrylhydrazyl (DPPH) radicals, while simultaneously enhancing the activity of endogenous antioxidant enzymes such as superoxide dismutase (SOD), catalase (CAT), and glutathione peroxidase (GSH-Px) in murine hepatic and serum samples (Wang X. et al., 2017). The red ginseng-Ophiopogon japonicus complex polysaccharide (SMP-AP) has been identified as a bioactive metabolite capable of mitigating oxidative stress-related cellular damage through modulation of cellular defense systems. In hydrogen peroxide (H2O2)-challenged IPEC-J2 cells, OJPS treatment was shown to significantly elevate total antioxidant capacity (T-AOC) and augment the activities of GSH-Px and SOD, thereby counteracting oxidative damage and reducing malondialdehyde (MDA) accumulation. Furthermore, OJPS was found to upregulate the expression of nuclear factor erythroid 2-related factor 2 (Nrf2) and its downstream antioxidant genes, conferring protection against H2O2-induced oxidative stress in HepG2 cells (Kang et al., 2023). Additional investigations have elucidated the protective effects of OJPS against 1-methyl-4-phenylpyridinium (MPP+)-induced cytotoxicity in PC-12 cells. These effects were mediated through cellular rejuvenation, suppression of apoptotic pathways, amelioration of oxidative and endoplasmic reticulum (ER) stress, restoration of mitochondrial function, and inhibition of the Notch signaling pathway (Liu and Li, 2018). Moreover, OJPS has been shown to activate the AMP-activated protein kinase (AMPK)/Nrf2/heme oxygenase-1 (HO-1) signaling axis, leading to enhanced expression of SOD, GSH-Px, and CAT mRNA, which collectively ameliorated exercise-induced hepatic injury in murine models (Ren et al., 2022). The relationship between extraction parameters and antioxidant efficacy was systematically investigated by Wang et al. (2018), who demonstrated that ultrasound-assisted extraction power significantly influences the antioxidant capacity of OJPS. Maximum radical scavenging activity was observed at an optimal ultrasonic power of 400 W, with antioxidant performance following a biphasic pattern of initial enhancement followed by attenuation as ultrasonic intensity increased. These findings suggest that the antioxidant potential of OJPS is closely associated with its molecular weight distribution and structural modifications, highlighting the importance of optimized extraction protocols for maximizing bioactivity.

Recent studies demonstrate that Ophiopogon japonicus tea enhances antioxidant capacity in paraquat-exposed *Caenorhabditis elegans*, extending lifespan while ameliorating age-related pharyngeal dysfunction and reducing lipofuscin accumulation (Yu et al., 2018). Furthermore, Ophiopogon-containing phytochemical formulations attenuate fibroblast senescence by suppressing p38 MAPK and p53/p21 pathways (Xiang et al., 2023). These findings indicate OJPS’s therapeutic potential for oxidative stress-related disorders.

### 4.3 Cardioprotective activity

**FIGURE 1:**
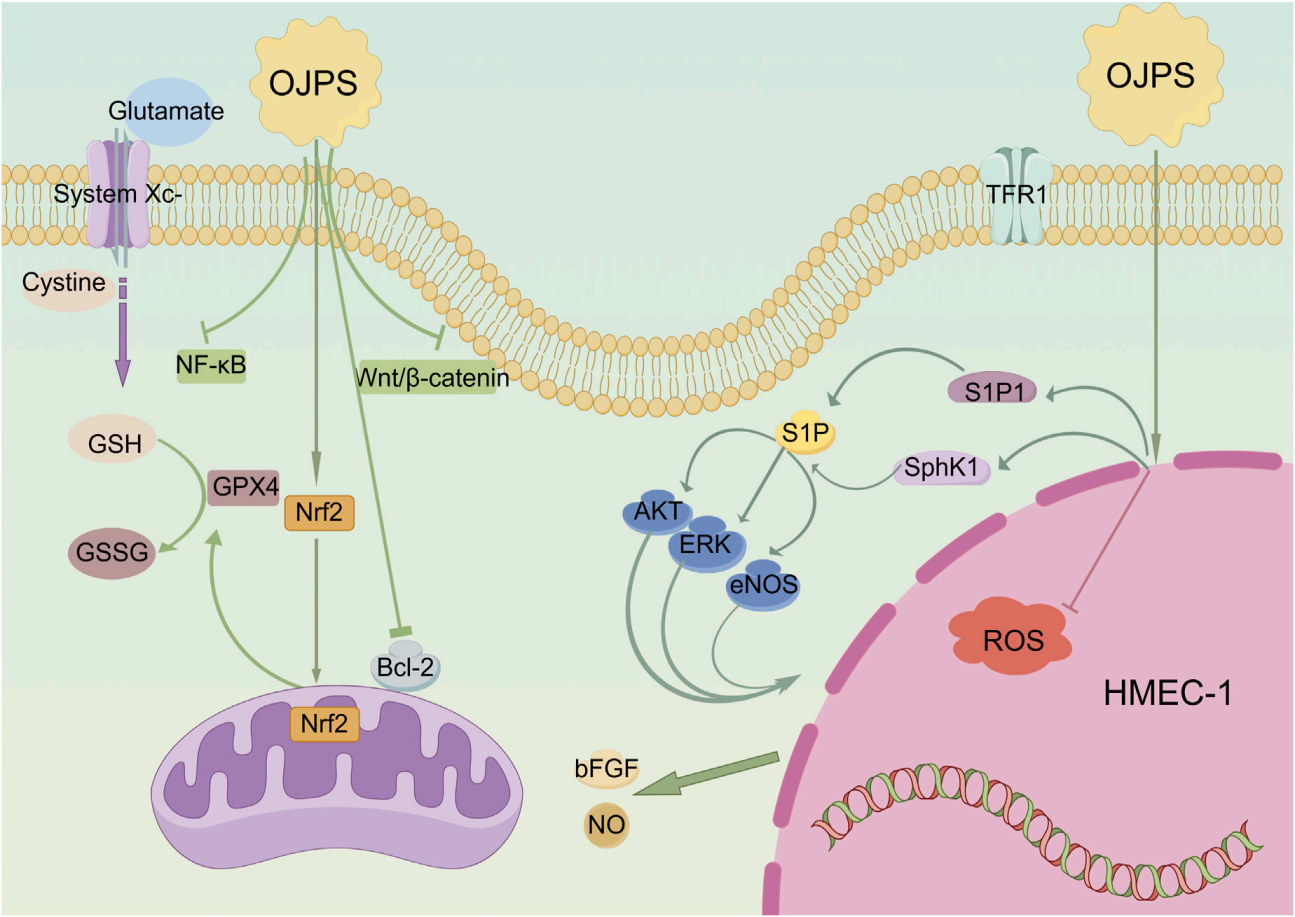
Diagram of the molecular mechanism of the Cardioprotective activity of OJPS. Created by Figdraw.

OJPS exhibits cardioprotective effects by enhancing cellular antioxidant defenses and maintaining cardiomyocyte viability. Doxorubicin (DOX)-induced cardiotoxicity, mediated through iron-dependent oxidative damage, was significantly attenuated by OJPS via Nrf2/GPX4 axis activation, as demonstrated by Chen et al. (2025). This mechanism effectively reduced iron-porphyrin complex accumulation in cardiac models. Further studies reveal OJPS protects vascular endothelial cells under oxidative stress. Li L. C. et al. (2017) reported OJPS mitigated H2O2-induced apoptosis and inflammation in HUVECs by regulating Bax/Bcl-2 ratio and suppressing caspase-3 activation. Similar protection was observed in brain microvascular endothelial cells, where OJPS alleviated high glucose-induced damage through NF-κB/COX-2 pathway inhibition (Gu et al., 2016). OJPS also demonstrates therapeutic potential in myocardial injury models. Chen et al. (2019) found OJPS enhanced TGF-β1-mediated cardiomyocyte survival while reducing ROS accumulation and suppressing Wnt/β-catenin and NF-κB signaling. In isoprenaline-induced cardiac injury, OJPS treatment normalized electrocardiographic parameters, decreased cardiac enzyme levels (AST, LDH, CK, CK-MB), and restored ATPase activity (Fan et al., 2020). These findings collectively establish OJPS as a multifunctional cardioprotective agent targeting oxidative stress and inflammatory pathways (Figure 1).

While extensive *in vitro* studies have demonstrated OJPS’s cardioprotective effects through multiple pathways, current *in vivo* investigations remain limited by the use of homogeneous animal models. This underscores the critical need for developing more diverse cardiovascular disease models to better evaluate OJPS’s therapeutic potential.

### 4.4 Immunomodulatory activity

OJPS has good immune-enhancing and stimulating effects (Liu et al., 2017). Meanwhile, OJPS can significantly enhance the immunization effect of the live Newcastle disease vaccine, indicating its synergistic immune-enhancing effect (Song et al., 2016). OJPS prepared as Ophiopogonan polysaccharide liposomes (OPL) can enhance the activation of macrophages, which can significantly improve their immune activity (Fan S. et al., 2015; Sun et al., 2016), and can also significantly enhance splenocyte proliferation as well as cytokine levels, antigen-specific antibody titers, and immune organ indices in OVA-immunized mice (Fan et al., 2016). OPL also enhances the immunoreactivity of KCs by activating the TLR4-NFκB signaling pathway, thereby regulating miR-1338 and miR-4796 and inhibiting cell apoptosis (Cui et al., 2022; Duan et al., 2021). In addition, OPL was able to regulate the expression of miR-14, which was able to significantly increase the phagocytic activity of KCs, promote the expression of iNOS and CD14, and reduce the level of apoptosis and secretion of ROS (Xing Xue et al., 2022). Ophiopogon japonicus inhibited proliferation, induced apoptosis, and inhibited the migration of NCI-H1299 cells (Liu et al., 2022). Ophiopogon japonicus extracellular polysaccharides (EPSs) reduce protease activity that inhibits the migration of gastric cancer cells, thus exerting antitumor activity (Xu et al., 2018). OJPS enhances the metabolic activity of macrophages by increasing the expression of MHC II, CD40, CD80, and CD86 (Lv et al., 2016).

Although the cardioprotective mechanisms of Ophiopogon japonicus polysaccharides (OJPS) have been extensively elucidated through *in vitro* studies, their therapeutic potential remains insufficiently characterized *in vivo* due to the predominant use of standardized animal models. This limitation highlights the necessity for establishing more sophisticated and clinically relevant cardiovascular disease models to comprehensively assess OJPS’s pharmacological efficacy.

## 5 Gut microbiota modulation effects

Ophiopogon japonicus has been extensively utilized as both a dietary supplement and a therapeutic agent to enhance human health and manage chronic diseases, primarily through modulation of the gut microbiota and maintenance of intestinal homeostasis. However, the systemic bioavailability of orally administered polysaccharides is restricted due to their high molecular weight and structural complexity, with most exerting bioactivity via gut microbial fermentation rather than direct intestinal absorption. Consequently, the interaction between polysaccharides and the gut microbiota is regarded as the predominant mechanism underlying their physiological effects (Gan et al., 2022).

The gut microbiota is dynamically influenced by diverse intrinsic and extrinsic factors, including geographical environment, dietary patterns, and host pathological states (Zhernakova et al., 2016; Gong et al., 2020). Alterations in microbial composition are driven by sustained chemical, physical, or biological stressors, such as chronic consumption of high-sugar/high-fat diets, irregular eating habits, and prolonged antibiotic exposure. OJPS exhibits broad-spectrum bioactivities, encompassing anti-inflammatory, antioxidant, antiviral, antitumor, and anti-aging properties, as well as modulatory effects on gut microbiota composition and intestinal immunity (Xue et al., 2024). Notably, OJPS has demonstrated efficacy and a favorable safety profile in preclinical models (Ye et al., 2023). Critically, OJPS serves as a promising therapeutic candidate for chronic diseases, primarily via remodeling gut microbial architecture, enhancing beneficial bacterial proliferation, and restoring microbiota-host metabolic crosstalk (Li et al., 2024a).

The gut microbiota is tightly regulated by host physiological processes and encodes a diverse repertoire of carbohydrate-active enzymes (CAZymes), which catalyze the depolymerization of dietary polysaccharides into absorbable monosaccharides (Holscher, 2017). Through microbial fermentation, indigestible polysaccharides are metabolized into SCFAs, such as acetate, propionate, and butyrate, which enhance intestinal nutrient absorption and serve as bioactive metabolites. SCFAs reduce luminal pH, establishing a mildly acidic colonic microenvironment that selectively promotes the proliferation of commensal probiotics (e.g., *Lactobacillus* and Bifidobacterium) while suppressing pathogenic colonization, thereby maintaining gut homeostasis. Additionally, SCFAs act as signaling molecules or systemic energy substrates via portal circulation, mediating both local mucosal immunity and extraintestinal physiological responses (Yue et al., 2022). Collectively, these findings underscore a symbiotic relationship between polysaccharides and gut microbiota, highlighting their profound implications for intestinal and systemic health.

### 5.1 Effect of OJPS on gut microbiota

Polysaccharides and gut microbiota maintain a symbiotic relationship characterized by bidirectional modulation (Huang et al., 2022). These compounds significantly influence both the phylogenetic composition and functional capacity of intestinal microbial communities, while gut microbiota mediate polysaccharide metabolism and subsequent host physiological effects. Microbial dysbiosis has been implicated in metabolic disorder pathogenesis, suggesting microbiota modulation represents a viable therapeutic strategy. Polysaccharides regulate intestinal microecology through two primary mechanisms: microbial fermentation of complex carbohydrate structures generates metabolites that support microbial growth and ecological stability, while their prebiotic activity selectively promotes beneficial taxa (e.g., *Bacteroides*, Roseburia) and inhibits pathogenic organisms, ultimately altering microbial diversity and functional potential (Wang et al., 2025).

**FIGURE 2:**
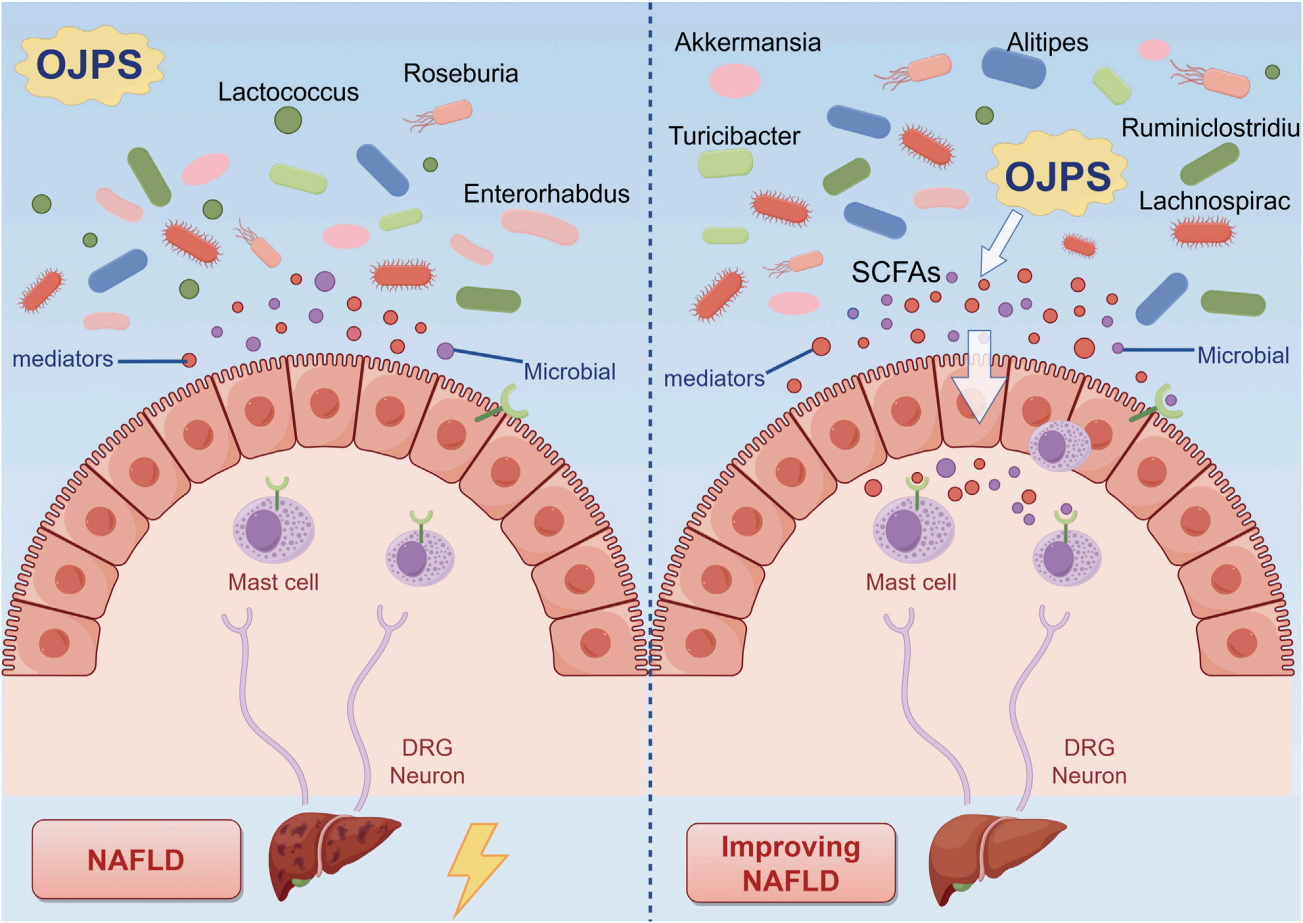
The interaction between OJPS and the gut microbiota. Created by Figdraw.

As demonstrated in Figure 2, Ophiopogon japonicus polysaccharides (OJPS) exhibit distinct prebiotic characteristics through selective enrichment of commensal bacterial populations, effectively modulating gut microbial phylogenetic composition and functional diversity to maintain intestinal homeostasis (Song et al., 2021). Functioning as preferential microbial substrates, OJPS fermentation significantly enhances beneficial genera (*Lactobacillus*, Bifidobacterium) while inhibiting pathogenic colonization. Quantitative analyses have confirmed OJPS-mediated increases in Bacteroidetes, Actinobacteria and Firmicutes populations, with concurrent suppression of *Enterococcus* and *Shigella* species. However, the molecular mechanisms governing these microbial interactions and their dynamic regulation remain to be fully elucidated, necessitating further investigation to establish robust therapeutic applications.

The red ginseng-Ophiopogon japonicus complex polysaccharide (SMP-NP) has been shown to stimulate *in vitro* proliferation of multiple *Lactobacillus* strains, particularly L. johnsonii BS15, while enhancing production of short-chain fatty acids including lactic acid and acetic acid, consequently acidifying the culture medium (Kang et al., 2023). In murine models, OJPS administration has been demonstrated to ameliorate high-fat diet-induced microbial dysbiosis by preserving Actinobacteria and Bifidobacteria populations while reducing fungal colonization (Wang S. N. et al., 2019). Furthermore, OJPS treatment effectively restored the physiological balance of Tenericutes and Bacteroidetes in obese mice, concurrently promoting beneficial genera (Alistipes, Ruminiclostridium, Rikenella) and suppressing pathogenic species (Lactococcus, Enterorhabdus, Turicibacter) (Wang X. et al., 2019). Notably, in non-alcoholic fatty liver disease models, OJPS supplementation has been found to enhance microbial diversity, with Akkermansia muciniphila identified as a key mediator capable of fermenting OJPS metabolites into acetate and propionate - metabolites inversely associated with disease progression (Zhang et al., 2022). However, the precise molecular mechanisms underlying A. muciniphila’s therapeutic effects require further investigation.

### 5.2 Effects of OJPS on intestinal epithelium

**FIGURE 3:**
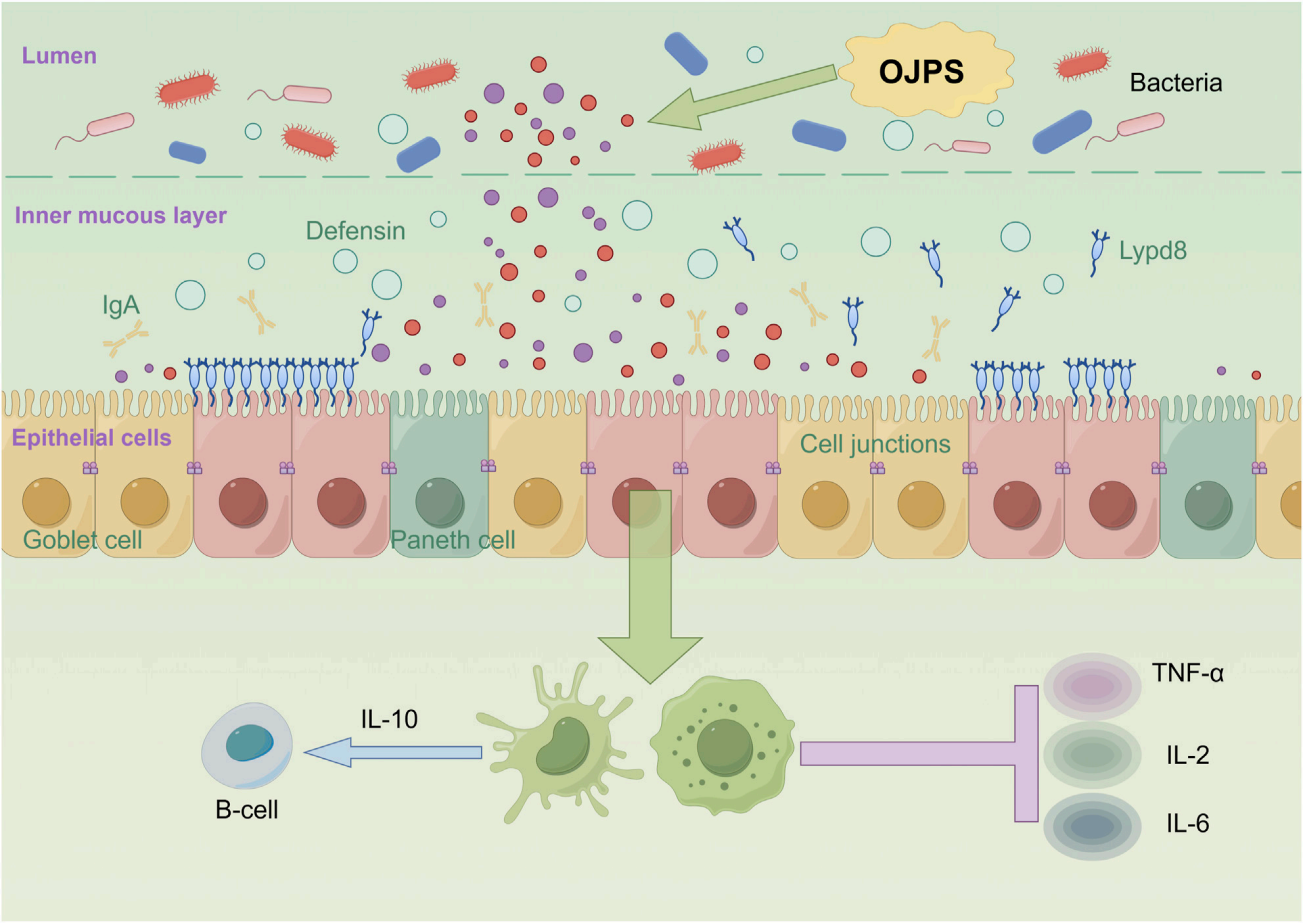
The interaction between OJPS and intestinal epithelium. Created by Figdraw.

The intestinal mucosal barrier is principally constituted by a monolayer of epithelial cells interconnected through tight junctions, which critically regulate paracellular permeability and establish an essential physical barrier (Zhang et al., 2021). As the predominant cellular constituents of this barrier, intestinal epithelial cells (IECs) actively maintain intestinal immune homeostasis via complex interactions with lamina propria cells (Liu et al., 2020; Xie et al., 2019). As depicted in Figure 3, OJPS has been shown to exert multifaceted protective effects on intestinal barrier function. These effects are mediated through the upregulation of tight junction protein expression, amelioration of mucosal immune dysfunction, and modulation of inflammatory mediators, collectively leading to enhanced barrier integrity and reduced intestinal permeability. Importantly, these beneficial effects are further potentiated by bioactive metabolites generated through microbial fermentation of OJPS.

Yuan and Ying (2017) found that Ophiopogon japonicus and Astragalus complex polysaccharides could attenuate the deleterious effects on the intestinal mucosal barrier by inhibiting pro-inflammatory factors (e.g., TNF-α, IL-2, and IL-6) and stimulating the release of anti-inflammatory factors (IL-10). Studies have shown that colitis can be alleviated by inhibiting inflammation-related factors and repairing the intestinal mucosal barrier (Li et al., 2022). An OJPS/chitosan/wheat protein (WP)-assembled nanoparticle, can effectively inhibit the production of NO and the expression of genes such as iNOS, COX2, TNF-α, CCL, etc., and thus attenuate the inflammatory response. OJPS nanocarriers are also effective in preserving the integrity of the intestinal epithelial barrier, preventing the damage caused by the inflammation in LPS-stimulated macrophages, and alleviating intestinal epithelial TJ barrier and permeability defects (Lin et al., 2020).

In addition, butyrate, a metabolite of the gut, significantly promotes the expression of tight junction proteins (e.g., cld-1, occludin, and ZO-1) in the ileum, thereby improving intestinal epithelial permeability (Peña-Rodríguez et al., 2022). In addition to stimulating the growth of beneficial bacteria through anaerobic fermentation of the intestinal microbiota, OJPS promotes the production of additional SCFAs in the gut. Therefore, it is hypothesized that OJPS protects the intestinal barrier by producing metabolites such as SCFAs.

Therefore, it is hypothesized that OJPS protects the intestinal barrier by producing metabolites such as SCFAs.

### 5.3 Antidiabetic activity mediated by gut microbiota and OJPS

**FIGURE 4:**
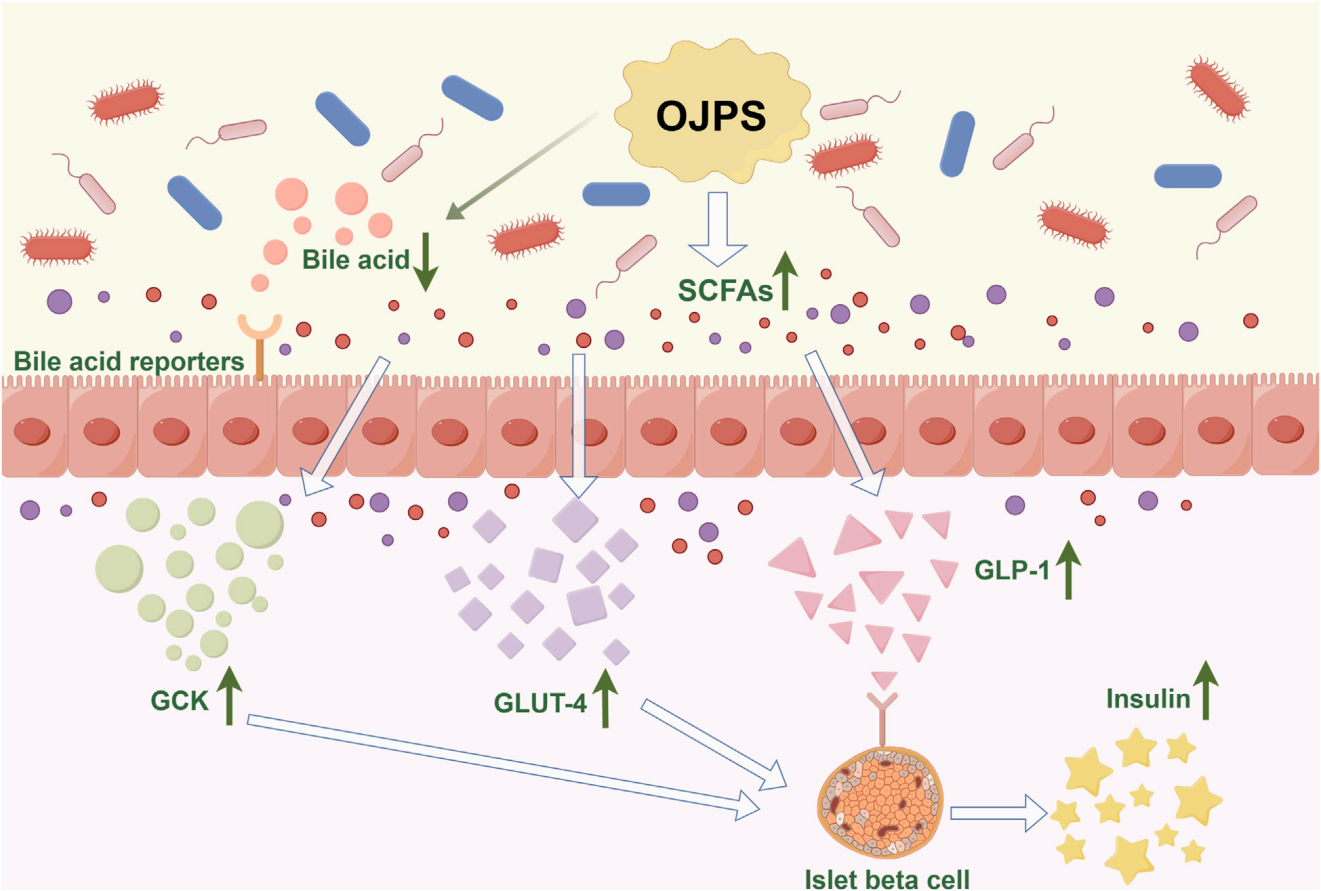
The Antidiabetic activity mediated by OJPS and gut microbiota. Created by Figdraw.

Type 2 diabetes mellitus (T2DM) is a common metabolic disorder characterized by insulin resistance and beta-cell hypoplasia. The occurrence of T2DM is associated with various factors such as human genetics, dietary habits, and exercise. Some studies have shown that an imbalance in the gut microbiota is one of the causative factors of T2DM (Wu et al., 2020). Studies have shown that the composition of the gut microbiota changes significantly before and after the onset of diabetes (Blandino et al., 2016). The relative abundance of intestinal *Lactobacillus*, Bifidobacterium, *Bacteroides*, Thick-walled phylum, *Clostridium*, and Ackermann’s bacilli decreases in patients with T2DM compared to normal subjects (Li et al., 2024b; Dong et al., 2025; Gurung et al., 2020), while the relative abundance of Rumatococcus, Brachybacterium, and *Clostridium* increases. The etiology of T2DM goes beyond changes in a single microorganism and is associated with the diversity of the gut flora and the balance of the gut flora. Food-assisted modulation of the gut microbiota for the prevention and treatment of diabetes is a new and viable approach (Figure 4).

Studies have shown that polysaccharides can regulate the composition of the gut microbiota, thereby improving diabetic blood glucose levels. Chinese botanical-based polysaccharides such as astragalus polysaccharide (Song et al., 2022), dendrobium polysaccharide (Chen et al., 2023), and mulberry huang polysaccharide (Ni et al., 2023) have been reported to ameliorate diabetes mellitus by improving the composition of the gut microbiota. ojps, which reduces the ratio of phylum Thickwiella/anabolic *bacillus* in C57BL/6 mice, alters the *lactobacillus Bacillus* spp. amino acid metabolism, and modulate abnormal gut microbiota (Shi et al., 2015). Meanwhile, OJPS can increase the number of intestinal probiotic flora such as *Lactobacillus* rhamnosus, *Lactobacillus* taiwanensis, *Lactobacillus* spp. and Bifidobacterium bifidum, and reduce the proliferation of harmful bacteria such as *Escherichia coli* and *Streptococcus* spp (Shi Lin-Lin, 2015; Yi, 2011).

Secondly, abnormal levels of SCFAs are one of the mechanisms that induce diabetes. Most polysaccharides cannot be directly digested and absorbed by the body, but are fermented by anaerobic bacteria in the gut to short-chain fatty acids SCFAs, the vast majority of which are located in the colon and absorbed by intestinal epithelial cells (Smith et al., 2013). Common SCFAs-producing bacteria include *Bacteroides*, Bifidobacterium, and *Streptococcus*. Abnormal SCFAs-producing bacteria can lead to abnormal levels of SCFAs (Karlsson et al., 2013). SCFAs can not only act directly on pancreatic β-cells to control their number and function, but also affect the transport of colon epithelial cells and accelerate the metabolism of colon epithelial cells (Ma et al., 2019). In addition, abnormal SCFAs lead to the release of inflammatory factors triggering intestinal inflammation, which impairs islet cell function and leads to insulin resistance (Takeuchi et al., 2023). Studies have shown that OJPS can restore gut microbiota homeostasis and increase the relative abundance of SCFAs-producing bacteria. OJPS, when degraded and utilized by the gut flora, increased the content of SCFAs and significantly increased the expression of the SCFA endogenous receptors G protein-coupled receptor (GPR) 41 and GPR 43. These produced SCFAs bind GPR and activate peroxisome proliferator-activated receptor (PPAR) γ, thereby limiting energy intake and downregulating blood glucose levels (Wang H. Y. et al., 2019).

In addition, the gut microbiota can alter the composition of bile acids and the activation of their receptors, thereby affecting the development of diabetes. Also, bile acids can inhibit the overproliferation of gut bacteria (Shi et al., 2016; Li X. et al., 2017). OJPS can adsorb and reduce bile acids in the intestinal lumen, affecting the metabolic synthesis of primary bile acids,and inhibiting their enterohepatic circulation. At the same time, the gut microbiota can produce a variety of metabolites that mediate the regulation of GLP-1 (Liang et al., 2024), which promotes insulin secretion and can play a key role in glucose metabolism. Some studies have shown that OJPS can significantly increase the expression level of GLP -1 in serum and upregulate the expression of GCK and GLUT4, thus enhancing insulin secretion and improving glucose metabolism in rats (Jia Yixin, 2023). Yunyun et al. found the hypoglycemic activity of OJPS in diabetic mice through the metabolome of feces. Hypoglycemic activity (Zhu et al., 2014) MDG-1 increased monosaccharide and succinate content, improved intestinal environment, inhibited intestinal glucose absorption and hepatic glucose catabolism, and induced the secretion of GLP-1 from L cells. Meanwhile, OJPS could also attenuate diabetes mellitus and diabetic nephropathy by decreasing 7H-pyridine and 20-deoxyglucoside. In addition, OJPS can significantly increase the activity of LXRα and then increase the transcriptional activity of CYP7A1, which accelerates the conversion of cholesterol to bile acids, i.e., OJPS can regulate the synthesis, secretion, and reabsorption of bile acids, and ultimately improve glucose tolerance and insulin resistance in mice (Wang Y. C. et al., 2017).

## 6 Discussions and future perspectives

OJPS, a principal bioactive metabolite derived from Ophiopogon japonicus, has garnered considerable attention owing to its extensive pharmacological properties. Recognized as a prominent traditional Chinese medicine with health-promoting effects, OJPS has emerged as a focal point in research pertaining to metabolic disorders and immunomodulation, attributable to its structural heterogeneity, multi-target bioactivity, and regulatory influence on gut microbiota. In this review, the advancements in OJPS research are systematically examined, encompassing its extraction methodologies, structural characterization, pharmacological mechanisms, and interactions with the gut microbiome. Furthermore, its potential therapeutic utility as a natural bioactive compound is elucidated, while the limitations of current investigations and prospective research directions are critically discussed.

Traditional investigations have predominantly centered on the direct pharmacodynamic effects of OJPS, including the amelioration of insulin resistance through the PI3K/Akt signaling pathway and the modulation of inflammatory responses via the TLR4/NF-κB pathway. However, emerging evidence suggests that the biological activity of OJPS may be intrinsically linked to its regulatory role in the gut microbiota-host metabolic axis. The production of SCFAs provides a mechanistic explanation for the systemic metabolic effects of OJPS despite its limited oral bioavailability, thereby substantiating the existence of a “microbiota-metabolite-host” axis. OJPS has been demonstrated to selectively enrich SCFA-producing bacterial taxa, such as Bifidobacterium and *Lactobacillus*, while suppressing the proliferation of endotoxin-secreting species (e.g., *Escherichia coli*), thereby enhancing the functionality of the microbiota-gut-brain axis. OJPS-derived metabolites have been implicated in interorgan communication. Butyrate, generated through OJPS fermentation, activates FFAR2 on intestinal L cells, stimulating GLP-1 secretion and improving insulin sensitivity. Conversely, propionate modulates energy homeostasis by crossing the blood-brain barrier, suppressing appetite-related neuropeptide expression in hypothalamic neurons. Furthermore, OJPS has been shown to attenuate high-fat diet-induced intestinal hyperpermeability by upregulating tight junction protein expression and enhancing mucin secretion, thereby mitigating systemic inflammation associated with endotoxin translocation.

OJPS represents a novel therapeutic approach that integrates traditional Chinese medicine with Western medical paradigms for diabetes mellitus treatment through intestinal microecological regulation. This strategy embodies the pharmacological advantages of a ‘multi-metabolite, multi-target’ intervention. Experimental studies have demonstrated that OJPS administration significantly ameliorates fasting glucose levels, insulin resistance, and dyslipidemia in diabetic model animals. These therapeutic effects were concomitant with restoration of gut microbial diversity and increased abundance of specific beneficial bacterial taxa. Notably, OJPS exhibits selective microbiota-modulating properties, particularly in suppressing the excessive proliferation of conditionally pathogenic bacteria. Subsequent metabolomic analyses revealed significant elevation in fecal SCFA levels, including butyrate and propionate, coupled with reduced endotoxin (LPS) concentrations in treated subjects. These findings strongly suggest that the amelioration of microbiota-host metabolic interactions constitutes the core mechanistic basis for OJPS-mediated therapeutic effects.

Current research on OJPS has achieved significant progress, yet several critical issues remain to be addressed through further investigation. The structural heterogeneity of OJPS has been identified as a potential determinant of its bioactivity, as functional variations have been observed among polysaccharide fractions derived from the same extraction batch. This underscores the necessity for comprehensive structure-activity relationship analyses. Furthermore, while the microbiota-modulating effects of OJPS have been preliminarily characterized, current investigations remain largely descriptive, focusing predominantly on compositional changes in microbial populations without elucidating the underlying metabolic pathways or establishing causal relationships. To advance understanding, integrated multi-omics approaches should be employed to systematically delineate the complex interplay between OJPS, gut microbiota, and host physiology. Such studies would facilitate identification of crucial regulatory nodes, whether specific microbial taxa or metabolic pathways, within the OJPS-microbiota-host network.

Recent advances in OJPS formulation strategies have demonstrated promising therapeutic applications. A notable study by Lin et al. (2022) developed chitosan/OJPS/casein hydrolysate (CS/OJPS/CL) co-assembled biodegradable nanoparticles, which were shown to significantly enhance the protective effects of OJPS against Ni2+-induced cytotoxicity and suppress LPS-stimulated nitric oxide production more effectively than OJPS alone. These findings suggest that polysaccharide-protein nanocomplexes may serve as efficient nanocarriers for oral delivery of bioactive polysaccharides. Furthermore, OJPS has been successfully employed to improve the biopharmaceutical properties of tetrandrine (THSG), including enhanced bioavailability and prolonged pharmacological activity (Sun et al., 2018). Collectively, these innovative approaches provide valuable insights for expanding the therapeutic applications of OJPS through advanced formulation technologies.

## 7 Conclusion

This investigation elucidates the intricate interactions between OJPS and gut microbiota, highlighting its potential pharmaceutical and nutraceutical applications. Current research progress in gut microbiota-disease correlations has enabled more precise characterization of specific microbial species and their metabolic byproducts. Distinct microbial signatures and associated metabolic profiles have been identified as potential diagnostic biomarkers. As a bioactive prebiotic agent, OJPS exhibits therapeutic efficacy in intestinal homeostasis maintenance through selective microbiota modulation, presenting novel intervention strategies for microbiota-related disorders while supporting overall host health.
